# Supplementary material for: Hebbian plasticity in parallel synaptic pathways: A circuit mechanism for systems memory consolidation
Source: PLoS Comput Biol. 2021 Dec 7;17(12):e1009681. doi: 10.1371/journal.pcbi.1009681 (PMC8683039; doi:10.1371/journal.pcbi.1009681)
Supplement: Attachment — Submitted filename: response to reviewers.pdf [file pcbi.1009681.s001.pdf]

Reviewer #1: This manuscript presents a well-designed and neatly executed study of systems-level memory consolidation in spiking and rate neural networks. The authors identify a circuit motif and a simple learning rule that allows copying of information between the synaptic weights of an indirect and a direct pathway. The manuscript is well written, clearly organized, and describes the results in an easily understandable fashion.

Response: We thank the reviewer for this very positive feedback! We nevertheless have reorganized the manuscript and have integrated the Supplementary Material in the main text, as suggested by Reviewer #2.

I have some questions and concerns, listed below:

The authors briefly review the literature in the introduction, but in addition it would be useful to have a paragraph that clearly states what the authors perceive as the ***main novelty or scientific advance*** *of the present work compared to other models of memory consolidation. Is it the fact the copying of information was achieved in a spiking model here? (This was also attempted in Tome et al: https://doi.org/10.1101/2020.12.22.424000). If so, it would seem appropriate to extend the study of spiking models beyond Figures 1 and 2. The discussion offers details on the relation of the present manuscript to previous phenomenological work, but* ***not much comparison to prior work in the neural networks/computational neuroscience literature****. More details on how the present copying mechanism is superior to or more biologically relevant than what has been done before would help.*

Response: The main novelty or scientific advance of the presented work is a mechanism for the transfer and the reorganisation of memories over time and across networks. In the Results, the suggested consolidation mechanisms were demonstrated to be independent of the level of modeling (integrate-and-fire, multicompartmental, or rate models). After an extensive reorganization of the manuscript (see below for details), there is now also a mathematical approach on this in the Results. A direct comparison of this new mechanistic framework of systems memory consolidation to other work is difficult because almost all other theories on this topic are more or less phenomenological (at various levels of complexity) and do not refer to the anatomical structure that imposes temporal delays and the synaptic plasticity of the underlying neural circuits. A notable exception is the work by Tomé, Sadeh, and Clopath (thanks to the reviewer for pointing us to the preprint), which we have added to our list of references and have included it in our Introduction and Discussion.

One aspect in which the present work appears to differ is the focus on the particular circuit motif the authors discuss, which is suitable for feedforward, hetero-associative memory, while many other papers on systems-level consolidation focus on recurrent, auto-associative memory systems. This may make this study potentially more relevant to motor skill or habit acquisition than to consolidation of declarative memories - see below.

Response: The reviewer correctly points out that our model is most suitable for feedforward, hetero-associative memory, which includes non-declarative forms of memory, but still embraces declarative memory. We mostly referred to declarative memories in the Introduction and the Results, but we also made links to other forms of memories in the Discussion in the section entitled "Consolidation of non-declarative memories''. To better outline from the start that we focus on feedforward network architectures (it is non-trivial to generalize our model to recurrent networks), we improved the Introduction; there we wrote: "We assume that such associations are stored in synaptic pathways between an input area — neurally representing the cue — and an output area — neurally representing the response." And now we have added: "Thus, our work relates to feedforward, hetero-assocative memory (and is thus applicable to both declarative and non-declarative memories) but does not directly address recurrent, auto-associative memory (see, e.g., Hopfield 1982, Zenke *et al.* 2015, Tomé *et al.* 2020).". We also changed the manuscript at various other points (e.g. in the Abstract and the Introduction) to better relate our work to procedural memories.

One major way in which the manuscript could be improved would be for the authors to show not merely that the copying of information is possible using their mechanism (which they do by monitoring the correlation of weights), but that this actually confers a computational advantage to a multi-stage memory system compared to a homogeneous memory system with the same number of synapses. In other words, when comparing memory systems of the same size (number of synapses), does breaking up the network into several subsystems and copying information between them actually increase the memory (recall) capacity? There are good theoretical arguments for why this should be the case (assuming the copying works well), and this should be easy to show at least for online learning with effectively binary weights (i.e., for strongly bimodal weight distributions), since it is known that such networks exhibit very poor (strongly sublinear) scaling of the memory capacity with system size. A more nontrivial (and interesting) test of the proposed mechanism would be to show that it can actually achieve a larger recall capacity than a homogeneous system of the same size that is well-equipped for good memory performance, namely one that uses sparse coding or complex synapses to achieve a favorable scaling of the memory capacity with system size.

Response: Showing that our heterogeneous multi-stage system has a larger memory capacity (more patterns or longer memory lifetime) than a homogeneous system of the same size (e.g. with the same number of synapses) is indeed interesting, but we do not expect new insights that go beyond the existing literature. In particular, there are already quite powerful theoretical results on this topic (e.g. by Roxin and Fusi, 2013), and we believe that they also apply to our system. In an extended Discussion (in "Theory requirements", (4)) we now explain this important relation of our model to earlier insights on memory capacity. We chose to not explicitly reproduce this behavior in the Results in order to maintain the focus of the manuscript on biologically plausible mechanisms for transferring and transforming (beyond merely copying) information across networks. Nevertheless, we also explicitly mention complex synapses (Discussion) and sparse coding (end of Results) in the revised manuscript.

(As an aside, my sense is that this manuscript really describes a mechanism rather than a conceptual advance, and personally I would hesitate to describe this as a "theory" in the scientific sense of the word. However, I understand that the word "theory" has been so frequently abused in the memory literature that the authors may feel that adding another "theory" to the list would do little harm.)

Response: Touché, maybe forgive us this minor decadence :). We indeed use the term "theory" mainly to compare our proposed mechanisms to those of related "theories" in the memory literature. To alleviate this wrong use of "theory" a bit, we replaced "theory" in many instances by other terms but kept the term "Parallel Pathway Theory".

In the hippocampal example of Figs. 1 and 2, it is not entirely clear what computational goal consolidation serves, or how the proposed scheme fits in which the many ideas in the literature about the different functions of the SC and perforant pathways. The underlying assumption appears to be that place cells are learned from entorhinal grid cell input (it's unclear if that's in agreement with the experimental literature), and that this learned mapping from grid to place cells is then copied from the SC pathway to the perforant path. If this is the case, it would appear to make some strong predictions about place cells representations. In particular, when an animal returns to an environment it has experienced a long time ago, one may encounter a situation in which there would be no place cells in CA3 (the environment has been forgotten in the SC pathway), but there are place cells in CA1 (the environment is remembered in the perforant path). Has such a situation ever been observed experimentally?

Response: The purpose of Figs. 1 and 2 (numbers in the old manuscript, now 1 and 3) is to illustrate the suggested transfer mechanism for systems memory consolidation. Whereas Fig.1 is more abstract and does not rely on a particular neurobiologically motivated representation, in old Fig. 2 (now 3) we turn to the specific example of place cell and grid cell representations in order to illustrate that the mechanism is robust to differing cue representations in the two pathways. We indeed implicitly assumed that place cell representations are generated from grid cell representations, even though this

is far from being settled. To better acknowledge this strong assumption and to indicate the current state of the art on place cells and grids cells, we added at the end of the third paragraph of the section "Consolidation of spatial representations" in the Results some text and refer to a classical paper on this topic (Solstad *et al*., 2006) and two recent reviews on place cells and grid cells (O'Keefe and Krupic, 2021; Tukker *et al*., 2021)."

Similarly in Fig. 3, it is encouraging that the authors can find model parameters that reproduce the behavioral effects of perforant path lesions. However, do we know experimentally whether in this (hippocampus-dependent) task the initial memory is in fact stored in the SC pathway, i.e., does an SC lesion shortly after task learning erase the memory trace? Conversely, does an SC lesion long after task learning leave the task performance unaffected, and preserve the CA1 place fields?

Response: In our model, we consider the CA3 region (including the Schaffer collateral (SC) pathway to CA1) as the key region for memory acquisition, but not for its long-term maintenance. This is motivated by two studies: Nakazawa *et al*. (2003) (already cited in our manuscript) showed that mice with NMDA receptor deletion restricted to CA3 pyramidal cells in adulthood are impaired in rapidly acquiring the memory of novel hidden platform locations in a delayed matching-to-place version of the Morris water maze task but are normal when tested with previously experienced platform locations. Moreover, Nakashiba *et al*. (2008) reversibly inhibited synaptic transmission at the SC pathway and found that it is required for rapid one-trial contextual learning but is dispensable for incremental spatial learning. We have added Nakashiba *et al*. (2008) to our list of references and now better indicate (at the beginning of Results, third paragraph of the section "A mechanistic basis for systems memory consolidation" and briefly also in the third paragraph of the section "Consolidation of place-object associations in multiple hippocampal stages", i.e., the text on Fig. 4 [old figure number was 3]) the neurobiological foundation of our assumption that memories are initially stored in the trisynaptic pathway involving CA3 and the SC pathway.

In Fig. 2F, is there a simple explanation for the dips in the PP_{CA1} tuning to the left and right of the peak?

Response: The reason for the dips next to the PP_{CA1} tuning is the finite range of frequencies of the input to the PP via the entorhinal grid cells. Given these, the best possible reconstruction of the place cell contains "ringing". This is akin to reconstructing a signal containing very high frequencies with a finite set of Fourier components, which also leads to ringing. In the fifth paragraph of the section "Consolidation of spatial representations", we added clarifying sentences to point this out to the readers of the document.

As for the multi-stage consolidation simulations, it is of course well-known that essentially any process that generates an exponentially decaying memory signal on a certain (adjustable) timescale can be replicated with different timescales, and when these different timescale processes are read out with appropriate weights the corresponding exponential decays can be superimposed to approximate a power law. The same appears to apply to the system studied by authors in Fig. 4, and by choosing the parameters (learning rates and relative readout weights) appropriately, the system would presumably be able to generate overall memory signals that decay as power laws with various exponents, or as any number of other functions of time that don't approximate power laws. Is there anything specific to the proposed system that would point to a particular preferred memory decay function, or allow a concrete prediction?

Response: The reviewer is of course right that other forgetting curves could be constructed by summing exponentials with different weights. We had to commit to one choice of learning rates and a readout strategy. For the learning rates, we chose a solution that links back to the literature and covers a broad range of time scales. For the readout, we tried to use one that makes as little assumptions as possible, by letting all pathways contribute on an equal footing. Taking the maximum over the pathways or the mean both generates a power law (see new sentences in the third paragraph of the section "Consolidation from hippocampus into neocortex by a hierarchical nesting of consolidation

circuits"). Given that we don't know which readout strategy is used by the brain when there are several memory traces, we would refrain from making any specific predictions here. Independent of the specific model choices, a requirement for power law forgetting is the presence of a broad range of time scales, which we assigned to the different pathways.

Can anything universal be said about the effects of lesioning one particular set of plastic synapses, which led to forgetting in Fig. 3, but could perhaps also be compensated for if nearby pathways have similar forgetting timescales?

Response: If we lesioned a set of synapses that covers a specific range of time scales such that there is a "gap" in the time scales that are present, we should see an impairment of consolidation if the lesion is done before the memory has "moved on". If there is a different pathway with a similar time scale, there should be very little effect. To include this thought in the manuscript, we have added a paragraph in the Results at the end of section "Consolidation of place-object associations in multiple hippocampal stages" on Fig. 4 (new number).

I understand that mechanistically the proposed multi-stage system requires appropriate time delays for the (very) indirect pathways in which the memory is initially stored, and one prediction is that the time delay for recall decreases during consolidation. This raises several questions: If the goal was in fact to eventually store information in a direct pathway with a short delay, why would the brain choose to initially store that information in an indirect pathway with a very long transmission delay? Is it reasonable to assume that achieving a short transmission delay is a central goal of memory consolidation? This may well be applicable to motor skill learning, or habit formation, but for the majority of the paper the authors frame their approach in terms of declarative memory. For episodic or semantic memory we don't usually think of recall from a cue as immediately initiating an associated action, but more commonly recalled episodes and semantic information have to first be combined with other sensory inputs to inform decision making before any reasoned action can be taken. How can we reconcile this?

Response: Very good point. We think that it is unknown if and why the brain may choose to initially store information in a pathway with a very long transmission delay (although this could be related to preprocessing for storage). Importantly, we believe that achieving a short transmission delay is NOT a central goal of memory consolidation. Our model suggests though that it may be a nice side effect in some cases, and this side effect should not be limited to declarative memories. To communicate this also to the readers of the manuscript, we have added a paragraph in the Results at the end of section "Consolidation from hippocampus into neocortex by a hierarchical nesting of consolidation circuits".

Finally, is the reduction in the delay of recall actually a necessary feature of systems-level consolidation? Clearly in the circuit motif the authors propose copying information using STDP works from the indirect to the direct pathway and not the other way around. However, it appears an "indirect" pathway in this study really just means a pathway with a longer transmission delay. As far as I can tell the authors imagine several layers of fixed weights interleaved with a single plastic layer in which memories are stored. The fixed weights are either not modeled at all, or simply modeled as identity transformations. Why would it have to be the case that the plastic layer in the indirect pathway is the last one, as in the SC pathway, or the middle one as in Fig. 4a? Could we not equally well make the first layer of the indirect pathway plastic and then introduce a delay by adding a layer of fixed weights afterwards? It would appear that copying information from such an indirect pathway to a direct one should work without problem at least if the fixed weights implement an identity transformation. However, in this situation the memory readout from the indirect and the direct pathways would actually have the same delay, since in both cases the memory resides in the first layer of weights after the input. If the speed of memory retrieval was a major concern, this would seem like a natural circuit to use.

Response: As already mentioned in our previous response, we believe that the reduction in the delay of recall is NOT a necessary feature of systems-level consolidation in general, but a natural consequence of the applied learning rule (STDP) that we have used on pathways with different delays. Regarding the storage of an initial memory in an indirect pathway in Fig. 1A and B (or between "Input 1" and hippocampus in Fig. 5), it is not important in which layers of the indirect pathway memories are acquired. We merely chose, for simplicity, to store our simple associations in a single layer of the indirect pathways (e.g. the second layer in SC, Fig. 1B). In principle, a more complex association can involve plasticity during acquisition in multiple layers of the indirect pathway, which would not affect our proposed transfer mechanism. For an iteration of the transfer mechanism, the constraint exists that the direct pathway of one transfer step is part of the indirect pathway of the next transfer step (e.g. PP-CA1 in Fig. 4 is the direct path of the first transfer step from EC → DG/CA3 → CA1 and is part of the indirect path EC → CA1 → SUB of the second transfer step). To make this clearer in the manuscript, we added a sentence after the first reference of Fig. 1F and a paragraph after equation (8) in the mathematical analysis.

Regarding the comment on a "single plastic layer", we indeed make the strong assumption that the initial memory acquisition is restricted to a single indirect pathway. However, in the brain during memory acquisition there might be some plastic changes at more (or all) levels. We speculate that this plasticity at other levels is much weaker (and assume for simplicity that it is absent in our model); the reasons why plasticity is different across levels are unknown (and how the existence of a schema could change this, as suggested in Tse et al. 2011). The assumption of decreasing plasticity along the hierarchy during memory acquisition may be related to the assumption of decreasing plasticity along the hierarchy during consolidation. However, neuromodulation could also be involved in the regulation of plasticity (see Discussion, second point of "Theory requirements and predictions"). To make our assumption on plasticity during acquisition more explicit and clear from the beginning on and explain that this simplification does not affect the proposed transfer of memories, we now say in the third paragraph of the Results (section "A mechanistic basis for systems memory consolidation") that we restrict memory acquisition to a single plastic layer (e.g. the Schaffer collaterals in Figs. 1, 3, and 4, or the hippocampus in Fig. 5).

Reviewer #2: The manuscript proposes a novel mechanistic theory of systems memory consolidation that spans multiple levels of detail, from single neurons to networks to multiple brain areas. The main idea of the theory is that original memories, stored in synaptic connections of one neural pathway (e.g. in the hippocampus), are progressively copied to shortcut neural paths up the neural hierarchy using standard hebbian synaptic plasticity rules. The functionality of the theory was demonstrated on the level of single spiking neurons, on the level of internal hippocampal networks modeled by populations of rate-based neurons and on the level of the whole brain in the context of classical phenomenological consolidation models. The proposed theory is shown to be consistent with experimental results in lesioned animals and with basic requirements of classical consolidation theories, and therefore it can provide a mechanistic basis for the earlier theories on the neuronal level providing a theoretical framework for testing and comparing them.

The strength of the proposed work is in the novelty, originality and depth of its primary proposal - the parallel pathway theory (PPT). Since the neuronal mechanisms underlying systems consolidation is a long-standing and important question in neuroscience, I believe that the proposed theory definitely deserves to be heard by a large audience of neuroscientists. The theoretical analysis is sound and rigorous.

Response: Many thanks!

However, a weak point of the paper, in my point of view, is that it is unnecessarily long and not optimally organised in terms of how the theory is demonstrated in simulations. While the main idea of the PPT is clear, it is presented using a number of different models (i.e. an integrate-and-fire model, a biophysical neuron model, and several rate-based models) and it is often not clear how all these models and their learning equations are related to each other. This diversity of models could have been justified by a unified underlying theoretical results, but the validity of analytical conclusions presented in the appendix for the simulations in the main text are not always clear. In other words, if the presented theory is primarily a computational theory, one would expect some theoretical statements to be valid for the whole range of models and simulations employed to illustrate how the theory works. In the manuscript, as it is written, it is not clear whether this is the case.

Response: We very much agree with the reviewer's general comment that the manuscript could be improved by a better organization and a closer connection of the theory and the numerical simulations of different levels of modeling. Briefly, we have moved most of the unifying Theoretical analysis from the Supplement to the Results and have connected them to the simulations. Further changes are described in detail below in the specific points of MAJOR ISSUES.

Another weak point is the lack of predictions from the proposed theory. How can this theory be experimentally verified? How is it possible to distinguish this theory from other competing theories? If this theory provides a mechanistic basis for classical phenomenological theories of systems consolidation, can it shed some light into the validity of generality of those classical theories? These issues should be addressed in the Discussion. More detailed comments are given below, more or less in the order of importance.

Response: We very much agree and will respond to this general comment in great detail below (point 4 in MAJOR ISSUES). Briefly, we added several further predictions to the manuscript.

MAJOR ISSUES

1. The simulations (fig.1) and the associated theoretical analysis form the basis for the rest of the paper, but their presentation lacks clarity and seems underdeveloped. The theoretical analysis, as well as predictions derived from it, are for some reason put in the Appendix, whereas in my opinion it constitutes the core of the proposed hypothesis. Indeed, the authors claim to "propose a novel mechanistic foundation of the consolidation process" (line 43) and the link between the proposed consolidation mechanism and linear regression is an important theoretical insight in this respect,

especially for this journal's audience. Therefore, it seems reasonable to move the Supplementary material, or at least some part of it including the Eq. 43, into the main text with the required assumptions and the corresponding prediction (lines 646-648, see also point 4 below).

Response: The manuscript was designed such that the main text is relatively short (the Results covered only 10 pages, including 4 figures+captions) without sophisticated math, and thus was accessible to a general audience. In the Results, the key points of the theory were demonstrated and illustrated in simulations that covered several levels. To nevertheless provide a mathematical framework that explains the underlying theory and connects these approaches, we provided a "Theoretical analysis" as a supplement, which was addressed to readers with sufficiently strong mathematical background. We separated the two approaches to not decrease the readability of the main text. However, we agree with the reviewer that it was often not clear enough how the theory is connected to simulations. To solve this problem and to emphasize the core of the proposed hypothesis, we moved, as suggested, the "Theoretical analysis" (former Sections 1-3, including the mentioned equation and the corresponding prediction) from the supplement to the beginning of the Results, and integrated this analysis in the main text. The former Section 4 of the "Theoretical analysis" became part of the Methods on the hierarchical consolidation.

2. It would be helpful to more directly link the theoretical model and results in Supp. sections 1 and 2 to the simulation results in Fig.1. In particular, how does the spiking neuron model in Methods map to the theoretical model and in what conditions they are equivalent? What are the equivalents of W, V, x and x', f and g of the theoretical model in the simulation? How do the simulation results in Fig.1 follow from the theoretical results (Eq. 43) ? Can they be directly compared in Fig.1?

Response: The above-mentioned reorganization of the manuscript greatly facilitated linking the mathematical approach to the simulations in Fig. 1. In the new Results section "Theory of spike timing-dependent plasticity (STDP) for parallel input pathways" we now refer back to the simulations in Fig. 1 several times. Also in the Results section "Consolidation of spatial representations" we now have many links to the theory. While the theory (former Eq. 43 is now Eq. 7) was derived for a linear model, the simulations in Fig. 1 for the integrate-and-fire model with Poisson input are clearly nonlinear. Nevertheless, basic results from the (linear) theory hold in that case. In the revised manuscript, we have explained the equivalents of W, V, x and x', f and g of the theoretical model in the simulation, which helps to connect the two approaches and to see that the core results do transfer from the theory to the simulations.

3. The model of Section 3 of the results seems overly complicated in order to show an intuitively appealing result in Fig. 3C (bottom panel). Moreover, the relation of this rate model to the rate model theoretically analysed in earlier sections is not clear. What is the relation between the learning equation (Eq. 40) for the rate model in the Supp. Section 1 and Eq. 24 for the rate model in Methods? Logically it should be the same model, but it is not clear from the text if that's the case. Do the theoretical results in Supp. Section 1 are applicable for the model of Fig.3 ?

Response: We agree that the model may seem overly complicated for the simple result in Fig. 4C (former Fig. 3C), but we wanted to design it such that it can be matched in a natural way to the experimental results in the water maze. In the revised manuscript, we explain in the Methods (new paragraph after Eq. 32) the relation of one learning equation (former Eq. 40 in Supplement, now Eq. 2 in Results) to the other learning equation (former Eq. 24 in Methods, now Eq. 32 in Methods). We hope that this helps to better see that the theoretical results are indeed applicable to the model in Fig. 4 (former Fig. 3).

It seems that it would be much easier to demonstrate consolidation/forgetting using the simulation setup in Fig.2 (i.e, memory of location in the linear track), but with the model of Fig.3A (or an equivalent rate model, e.g. that in the Supp. section 1). The introduction of object-place associations and two-dimensional grid-cells and place-cell codes seems unnecessary and makes the paper

considerably longer, while it is not helpful to understand neither the Fig.3C (bottom panel) nor reproduce the experimental data in Fig. 3D.

Response: You are right that some of the features of the model in Fig. 4 (former Fig. 3) could be omitted, specifically the object codes in CA1 and SUB (but not in EC & CA3) and the place codes in EC and CA3 (but not in CA1 & SUB). In principle, one could also simplify the model to a cyclic 1D variable with 4 segments. However, the simplified model would have the same number of free parameters, and the mapping to the water maze would not be as intuitive. We would therefore prefer to keep the example simulations on memory of location in the linear track in Fig. 3 (former Fig. 2) and object-place associations in Fig. 4 (former Fig. 3) as they are. Having now integrated the theory at the beginning of the Results, it might be easier to perceive these simulations as evidence for the generality of the theory in more complex settings.

4. Are there experimentally testable predictions from the proposed theory, e.g. on the level of hippocampal networks, apart from the prediction mentioned in the Appendix (lines 646-648) ?

Response: The prediction that was mentioned in the Supplement (former lines 646-648) is now part of the Discussion in the section "Theory requirements and predictions, (1)" and has been adapted. Furthermore, in the section "Consolidation of place-object associations in multiple hippocampal stages" we inserted a paragraph on the predicted outcome of lesioning other plastic pathways (this was already briefly mentioned in the Discussion in "Limitations of the model and future directions"). Moreover, in the Results section "Consolidation from hippocampus into neocortex by a hierarchical nesting of consolidation circuits" we now discuss the predicted consolidation-mediated decrease of the time to recall. Finally, the predictions formerly in the caption of the Supplementary Fig. 2 B, i, ii, and iii are now part of the Results (new Fig. 2).

We also interpret some points in the Discussion (section "What limits systems memory consolidation") as specific predictions: a consolidation related transfer of memories fails if *"a shortcut pathway may not be present anatomically, or it may not host an appropriate representation for a given cue-response association in question"* or if *"shortcuts with simpler representations of cues and/or responses [...] do not allow a linear separation of the associations.*" Finally, also the four "Theory requirements" in the Discussion can be regarded as predictions, and we thus changed the title of this section to "Theory requirements and predictions".

Is there any evidence in the hippocampal replay literature supporting the prediction (lines 646-648) ? In one paper (Olafsdottir et al 2016, Nat Neurosci 19, 792–794 https://doi.org/10.1038/nn.4291), the replay in the EC was delayed with respect to CA1 by 11ms, which does not seem to be consistent with the model requirement.

Response: Ólafsdóttir *et al.* (2016) investigated the correlation of replay in the deep layers of the EC and CA1, and the observed delay is consistent with the known anatomy (CA1 projects via the subiculum to deep layers of the EC) and also consistent with our model requirements. In our Figs. 1B and 4A (new number) we do not mention deep and superficial layers of EC but we had this organization in mind when we connected inputs and outputs of EC to different parts of EC. The model predictions (formerly in lines 646-648, now part of the Discussion in the section "Theory requirements and predictions, (1)") are supported by O'Neill *et al.* (2017) who found a weak but significant coherence between replay events in superficial MEC and hippocampus. Even though the title of the O'Neill paper suggests that replay in EC and hippocampus is independent, this interpretation is biased by their choice to detect replay events in EC independently of hippocampus; in contrast, Ólafsdóttir *et al* detected replay events in the hippocampus, and found coordinated grid and place cell replay. A nice summary of this controversy can be found at https://www.peyrachelab.com/?p=190, and we now mention this issue briefly in the Discussion ("Theory requirements and predictions, (1)").

It seems that the theory makes it possible to formulate necessary conditions on time delays and activity correlations between direct and indirect pathways that are necessary to implement

consolidation (see e.g. caption of Supp.Fig2 B, i, ii and iii). It seems possible to experimentally verify all the three conditions for consolidation, unless the related data are already available in the literature. These issues can be addressed in the Discussion.

Response: The predictions that were formerly phrased in the caption of the Supplementary Fig. 2 B, i, ii, and iii are now part of the Results (see new Fig. 2B) and the Discussion ("Theory requirements and predictions"). Apart from results by Olafsdottir *et al*. (2016) and O'Neill *et al*. (2017) on correlations (see also the previous response), the already cited literature on STDP and the typical delays in neuronal systems support the predictions.

MINOR ISSUES

5. It is not clear whether the authors use the hippocampal formation just as a suitable example network to illustrate the functioning of their theory, or they argue for the specific role of the trisynaptic pathway in memory consolidation? Would it be possible to consolidate CA3 memories directly to EC-SUB connections, bypassing PP-CA1 ?

Response: The hippocampal formation inspired this study because it is clearly involved in memory consolidation and shows shortcut connections. The basic mechanism, however, is not restricted to this circuit. The reviewer is absolutely right that a direct consolidation from CA3 to PP-SUB would be possible if the memory lifetimes are similar enough. This issue is now addressed in the Results in a new paragraph at the end of the section "Consolidation of place-object associations in multiple hippocampal stages". The hippocampus is a suitable example but our theory is more general, and we hope that this is clearer after having moved the theoretical approach from the Supplement to the beginning of the Results.

6. Direct EC-CA1 connections are sufficient to create CA1 place cells (Brun et al (2002). Place Cells and Place Recognition Maintained by Direct Entorhinal-Hippocampal Circuitry. Science, 296(5576), 2243–2246. https://doi.org/10.1126/science.1071089). Do these results invalidate the model ?

Response: We think that the results by Brun *et al* (2002) do not invalidate the model but that they rather support it. While we assumed in the model for clarity that PP-CA1 is not plastic during memory acquisition, we do not exclude this possibility. Our memory-transfer model does not rely on specific pathways being the initial storage site. However, the plasticity may be weaker in the direct PP-CA1 compared to the indirect pathway, which motivates a slow transfer of memories.

To understand why the direct PP-CA1 connections could be sufficient to create CA1 place cells according to the Brun *et al* (2002) paper, we need to dig a little bit deeper. In the Methods of the paper by Brun *et al.* it is stated that "*Recordings began when the rat covered the whole surface of the open field and ran continuously on the track, usually after 3-7 days of training.*" This may be enough time for the PP-CA1 pathway to slowly acquire tuning such that place fields in CA1 emerge even without supervision from the SC pathway. However, even though there are place fields in CA1, Fig. 4 in Brun *et al.* show that rats with lesions in CA3 have an impaired memory recall in the Morris water maze, indicating that CA3 and the SC are important for memories beyond spatial representations.

The view of differential plasticity in the pathways (SC vs. PP-CA1) is motivated by two further studies (see also the related comment by Reviewer #1): Nakazawa *et al.* (2003) (already cited in our manuscript) showed that mice with NMDA receptor deletion restricted to CA3 pyramidal cells in adulthood are impaired in rapidly acquiring the memory of novel hidden platform locations in a delayed matching-to-place version of the Morris water maze task but they are normal when tested with previously experienced platform locations. Moreover, Nakashiba *et al.* (2008) reversibly inhibited synaptic transmission at the Schaffer collateral pathway and found that it is required for rapid one-trial contextual learning but is dispensable for incremental spatial learning. We have added Nakashiba *et al.* (2008) and Brun *et al.* (2002) to our list of references and now better indicate (at the beginning of Results, in the new third paragraph of the section "A mechanistic basis for systems memory consolidation" and briefly in the third paragraph of the section "Consolidation of place-object associations in multiple hippocampal stages" the neurobiological foundation of our

assumption that memories are initially stored in the trisynaptic pathway involving CA3 and the Schaffer collaterals.

7. I do not see why the results section describes the hierarchical model in Fig 4, while the theoretical results are obtained for the model in Supp. Fig.3 ? Why not directly use the model in Supp.Fig 3?

Response: Yes, we could have used the theoretical results directly, but the theory (now in the Methods section "Theoretical analysis of hierarchical consolidation") does contain a few simplifying assumptions, such as the exponential memory decay in the HPC that is put in by hand rather than by overwriting; and the theory does not incorporate technical aspects such as weight normalizations, which — as the reviewer probably knows — can easily change the learning dynamics. An instantiation of the theory in a computational model therefore strengthens the claim in our opinion. We therefore kept in the Results the model on "Consolidation from hippocampus into neocortex by a hierarchical nesting of consolidation circuits" including Fig. 5 (formerly Fig. 4). We also better motivated the "Theoretical analysis of hierarchical consolidation" and the related Fig. 6, which are now in the Methods (in the old version, this was in the Supplement and in the related Suppl. Fig. 3). More precisely, in the first paragraph that describes this analysis we compare it with the model used for Fig. 5 (formerly Fig. 4) in the Results.

8. Is there any evidence for "exponentially decreasing learning rates with distance from the hippocampus" (line 227), that seems to be a necessary condition for explaining the data by Wixted, 2004?

Response: We think that there is no empirical evidence for exponentially decreasing learning rates, even though it is a common assumption in systems consolidation models (e.g. in Roxin and Fusi, 2013 or in Tomé *et al*., 2020) and in synaptic consolidation models (Fusi *et al*., 2005; Benna and Fusi, 2016). We regard the systematic decrease and broad range (rather than a perfect exponential decrease) in learning rates within the consolidation hierarchy as a model prediction (see also Discussion, "Theory requirements and predictions " number 4).

9. The authors mention several other models of synaptic memory consolidation (line 274). How does the proposed model compare to them? What are the distinguishing characteristics between the models? Is it possible to envisage experimental tests to see which model is more plausible?

Response: We are not looking at synaptic memory consolidation, but systems memory consolidation. Clearly the two are not mutually exclusive. To better distinguish the two approaches, we have improved the respective paragraph in the Discussion (end of section "Theory requirements and model predictions"), mentioning now why they are conceptually related but nevertheless different.

10. Line 157, the reference to Morris and Lecar, 1981 is incorrect.

Response: Done.

11. Typo, line 48 : thec -> the
Typo: In practise -> in practice (throughout the text)

Response: These and many more typos have been corrected.

Further changes:

- We have added an "Author summary".
- We have included the supplementary Figure 1 now as part F of Figure 1.
- The Methods and the Results section "Theory of spike timing-dependent plasticity (STDP) for parallel input pathways" now use a uniform style and notation.
- We have adopted the PLoS CB style (latex template) of the manuscript.
- Several new references have been added.
